# Redetermination of 1-naphthalene­acetic acid

**DOI:** 10.1107/S1600536808036246

**Published:** 2008-11-13

**Authors:** Zhi-An Li, Dao-Yong Chen, Li-Jian Liu

**Affiliations:** a Key Laboratory of Oral Biomedical Engineering, Ministry of Education, School & Hospital of Stomatology, Wuhan University, Wuhan 430079, People’s Republic of China; b College of Chemistry and Molecular Science, Wuhan University, Wuhan 430072, People’s Republic of China

## Abstract

The crystal structure of the title compound, C_12_H_10_O_2_, was originally determined by Rajan [*Acta Cryst*. (1978). B**34**, 998–1000] using intensity data estimated from Weissenberg films. This redetermination provides a structure with significantly improved precision with respect to the geometric parameters. In the crystal structure, inter­molecular O—H⋯O hydrogen bonds, weak C—H⋯O hydrogen bonds and C—H⋯π inter­actions link the mol­ecules into a two-dimensional sheet lying parallel to (100).

## Related literature

For the original structure determination, see: Rajan (1978 ▶). For a description of the Cambridge Structural Database, see: Allen (2002 ▶); Bruno *et al.* (2002 ▶).
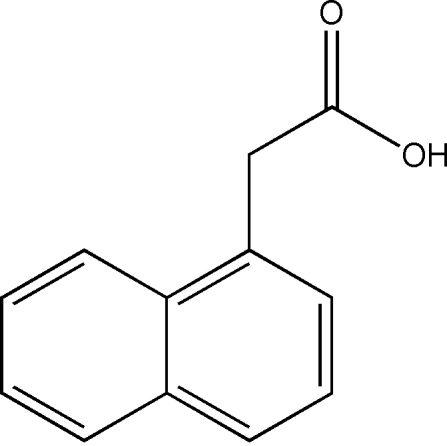


## Experimental

### 

#### Crystal data

C_12_H_10_O_2_
*M* *_r_* = 186.20
Monoclinic, 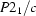

*a* = 12.7079 (19) Å
*b* = 5.1464 (8) Å
*c* = 15.014 (2) Å
β = 91.987 (3)°
*V* = 981.3 (2) Å^3^
*Z* = 4
Mo *K*α radiation
μ = 0.09 mm^−1^
*T* = 200 (2) K
0.20 × 0.04 × 0.02 mm

#### Data collection

Bruker SMART APEX CCD area-detector diffractometer
Absorption correction: multi-scan (*SADABS*; Sheldrick, 1997 ▶) *T*_min_ = 0.973, *T*_max_ = 0.993
9953 measured reflections
2025 independent reflections
1416 reflections with *I* > 2σ(*I*)
*R*_int_ = 0.023

#### Refinement

*R*[*F* ^2^ > 2σ(*F* ^2^)] = 0.049
*wR*(*F* ^2^) = 0.148
*S* = 1.04
2025 reflections
130 parameters
H atoms treated by a mixture of independent and constrained refinement
Δρ_max_ = 0.19 e Å^−3^
Δρ_min_ = −0.14 e Å^−3^

### 

Data collection: *SMART* (Bruker, 2001 ▶); cell refinement: *SAINT-Plus* (Bruker, 2001 ▶); data reduction: *SAINT-Plus*; program(s) used to solve structure: *SHELXS97* (Sheldrick, 2008 ▶); program(s) used to refine structure: *SHELXL97* (Sheldrick, 2008 ▶); molecular graphics: *PLATON* (Spek, 2003 ▶); software used to prepare material for publication: *PLATON*.

## Supplementary Material

Crystal structure: contains datablocks global, I. DOI: 10.1107/S1600536808036246/lh2723sup1.cif

Structure factors: contains datablocks I. DOI: 10.1107/S1600536808036246/lh2723Isup2.hkl

Additional supplementary materials:  crystallographic information; 3D view; checkCIF report

## Figures and Tables

**Table 1:** Hydrogen-bond geometry (Å, °)

*D*—H⋯*A*	*D*—H	H⋯*A*	*D*⋯*A*	*D*—H⋯*A*
C3—H3⋯O2^i^	0.93	2.61	3.541 (2)	177
O1—H1⋯O2^ii^	0.93 (3)	1.76 (3)	2.6723 (17)	168 (3)
C11—H11*B*⋯*Cg*1^iii^	0.97	2.87	3.746 (2)	151

Symmetry codes: (i) 
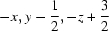
; (ii) 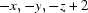; (iii) 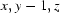. *Cg*1 is the centroid of ring C1/C6–C10.

**Fig. 1.**
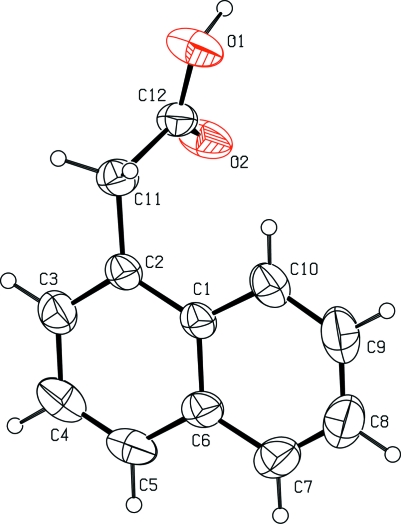
Molecular structure of (I), showing the atom-numbering scheme. Displacement ellipsoids are drawn at the 30% probability level.

**Fig. 2.**
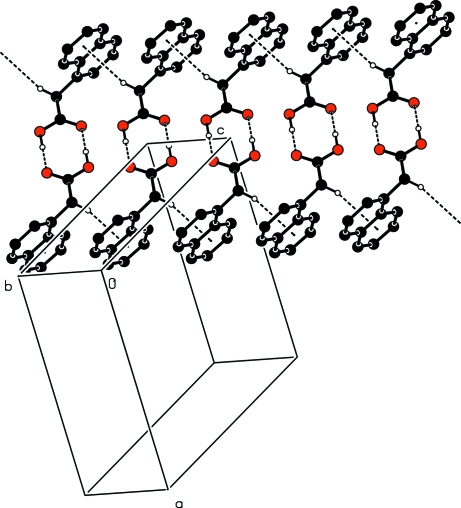
Part of the crystal structure of (I), showing the formation of the one-dimensional chain structure running parallel to the [010] direction formed by O—H···O and C—H···π interaction shown as dashed lines. For the sake of clarity, H atoms not involved in the motif have been omitted from the drawing.

**Fig. 3.**
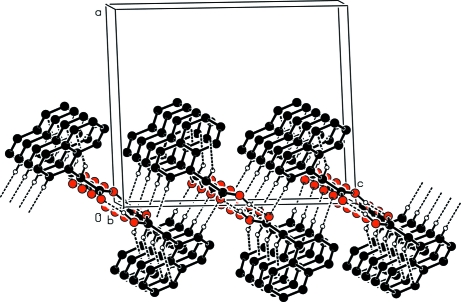
Part of the crystal structure of (I), showing the formation of the two-dimensional sheet parallel to the (100) plane formed by O—H···O, weak C—H···O hydrogen bonds and C—H···π interaction shown as dashed lines.

## supplementary crystallographic information

### Comment

A search of the Cambridge Structural Database (CONQUEST Version 1.10, CSD version 5.29, Allen, 2002, Bruno *et al.*, 2002) reveals that the structure of the title compound (I) was first reported (Rajan, 1978) with *R* = 0.129 for 776 observed reflections. However, the published report did not identify any supramolecular aggregation beyond the formation of a hydrogen-bonded dimers. We have now taken the opportunity to redetermine the structure of the title compound using data collected at 200 K.

In (I), we find the same phase at 200 K as those previously reported at ambient temperature. During the refinement of (I), we have refined the structure without any constraints, and the current precision is significantly better than those reported previously. Thus, for example, the previously reported s.u. values for the C—O bonds are 0.01 (Rajan, 1978); whereas from the present refinement of (I), these s.u. values are only 0.002. In addition, the *R* value is very much lower for the present refinement (0.0488). The dihedral angles between the naphthalene-ring plane (C1 to C10) and the carboxyl plane (C11/C12/O1/O2) are 80.6 (1)° (Fig.1) for the title compound and 81.3 (1)° for the original detemination, respectively. No unusual molecular features are worthy of discussion.

In compound (I), the molecules are linked by a combination of O—H···O, weak C—H···O hydrogen bonds and C—H···π interaction, into a two-dimensional network. In more detail, the supramolecular aggregation can be analyzed in therms of three aspects. First, the O1 atom in the molecule at (*x*, *y*, *z*), act as the hydrogen-bonding donor, *via* H1 atom, to the O2 atom in the molecule at (-*x*, -*y*, 2 - *z*), forming a discrete hydrogen-bonding dimer (Fig.2). Secondly, atom C11 at (*x*, *y*, *z*) acts as hydrogen-bond donor (Table 1) to the C1/C6—C10 aryl ring at (*x*, *y* - 1,*z*), forming a C—H···π interaction, which linked the dimers into a one-dimensional chain running parallel to the [010] direction (Fig.2). Finally, these adjacent [010] chains are linked together by a weak C3—H3···O2 hydrogen bond [C···O=3.540 (2) Å, symmetry code: -*x*, -1/2 + *y*, 3/2 - *z*), forming the final two-dimensional sheet lying parallel to the (100) plane (Fig.3). No other direction-specific interactions are observed between the neighbouring sheets.

### Experimental

1-Naphthalene-acetic acid (I), was obtained unexpectedly by reaction of mixing 2:1:1 equivalent molar amount of (I), 4,4'-bi-pyridine and Mn(ClO_4_)_2_.2(H_2_O) in 95% methanol (20 ml). The mixture was stirred for 30 minutes at 330 K and then filtered. Colorless needle crystals of (I) suitable for single-crystal X-ray diffraction analysis were grown at the bottom of the vessel in two weeks after slow evaporation of the solution.

### Refinement

All H atoms bonded to C atoms were located in difference maps and then treated as ring with C–H = 0.93 Å(aromatic), 0.97 Å (methylene) and refined in a riding mode [*U*_iso_(H) = 1.2*U*_eq_(C)]. H1 atom was found in the difference map and the O—H distance was refined freely [the refined distances are given in Table 1; *U*_iso_(H) = 1.5*U*_eq_(O)].

### Crystal data

**Table:**

C_12_H_10_O_2_	*F*_000_ = 392
*M**_r_* = 186.20	*D*_x_ = 1.260 Mg m^−^^3^
Monoclinic, *P*2_1_/*c*	Mo *K*α radiation λ = 0.71073 Å
Hall symbol: -P 2ybc	Cell parameters from 2268 reflections
*a* = 12.7079 (19) Å	θ = 2.7–24.9º
*b* = 5.1464 (8) Å	µ = 0.09 mm^−^^1^
*c* = 15.014 (2) Å	*T* = 200 (2) K
β = 91.987 (3)º	Needle, colorless
*V* = 981.3 (2) Å^3^	0.20 × 0.04 × 0.02 mm
*Z* = 4	

### Data collection

**Table:**

Bruker SMART APEX CCD area-detector diffractometer	2025 independent reflections
Radiation source: fine focus sealed Siemens Mo tube	1416 reflections with *I* > 2σ(*I*)
Monochromator: graphite	*R*_int_ = 0.023
*T* = 200(2) K	θ_max_ = 26.5º
0.3° wide ω exposures scans	θ_min_ = 1.6º
Absorption correction: multi-scan(SADABS; Sheldrick, 1997)	*h* = −15→15
*T*_min_ = 0.973, *T*_max_ = 0.993	*k* = −6→6
9953 measured reflections	*l* = −18→18

### Refinement

**Table:**

Refinement on *F*^2^	Secondary atom site location: difference Fourier map
Least-squares matrix: full	Hydrogen site location: inferred from neighbouring sites
*R*[*F*^2^ > 2σ(*F*^2^)] = 0.049	H atoms treated by a mixture of independent and constrained refinement
*wR*(*F*^2^) = 0.148	*w* = 1/[σ^2^(*F*_o_^2^) + (0.0798*P*)^2^ + 0.0818*P*] where *P* = (*F*_o_^2^ + 2*F*_c_^2^)/3
*S* = 1.04	(Δ/σ)_max_ < 0.001
2025 reflections	Δρ_max_ = 0.19 e Å^−^^3^
130 parameters	Δρ_min_ = −0.14 e Å^−^^3^
Primary atom site location: structure-invariant direct methods	Extinction correction: none

### Special details

**Table:**

Geometry. All e.s.d.'s (except the e.s.d. in the dihedral angle between two l.s. planes) are estimated using the full covariance matrix. The cell e.s.d.'s are taken into account individually in the estimation of e.s.d.'s in distances, angles and torsion angles; correlations between e.s.d.'s in cell parameters are only used when they are defined by crystal symmetry. An approximate (isotropic) treatment of cell e.s.d.'s is used for estimating e.s.d.'s involving l.s. planes.
Refinement. Refinement of *F*^2^ against ALL reflections. The weighted *R*-factor *wR* and goodness of fit *S* are based on *F*^2^, conventional *R*-factors *R* are based on *F*, with *F* set to zero for negative *F*^2^. The threshold expression of *F*^2^ > σ(*F*^2^) is used only for calculating *R*-factors(gt) *etc*. and is not relevant to the choice of reflections for refinement. *R*-factors based on *F*^2^ are statistically about twice as large as those based on *F*, and *R*- factors based on ALL data will be even larger.

### Fractional atomic coordinates and isotropic or equivalent isotropic displacement parameters (Å^2^)

**Table:**

	*x*	*y*	*z*	*U*_iso_*/*U*_eq_	
C1	0.27991 (11)	0.1115 (3)	0.80540 (10)	0.0549 (4)	
C2	0.19500 (12)	−0.0420 (3)	0.77187 (10)	0.0579 (4)	
C3	0.15963 (15)	−0.0059 (4)	0.68586 (12)	0.0769 (5)	
H3	0.1042	−0.1073	0.6636	0.092*	
C4	0.20412 (19)	0.1783 (5)	0.63049 (13)	0.0905 (6)	
H4	0.1787	0.1970	0.5720	0.109*	
C5	0.28320 (17)	0.3281 (4)	0.66118 (13)	0.0815 (6)	
H5	0.3118	0.4516	0.6238	0.098*	
C6	0.32383 (12)	0.3020 (3)	0.74899 (12)	0.0649 (5)	
C7	0.40692 (17)	0.4581 (4)	0.78251 (17)	0.0924 (7)	
H7	0.4355	0.5838	0.7460	0.111*	
C8	0.44567 (19)	0.4292 (6)	0.8661 (2)	0.1132 (9)	
H8	0.5007	0.5345	0.8870	0.136*	
C9	0.40414 (19)	0.2431 (6)	0.92165 (16)	0.1053 (8)	
H9	0.4319	0.2244	0.9794	0.126*	
C10	0.32338 (15)	0.0874 (4)	0.89291 (12)	0.0775 (5)	
H10	0.2965	−0.0361	0.9312	0.093*	
C11	0.14486 (15)	−0.2410 (3)	0.82983 (12)	0.0728 (5)	
H11A	0.0989	−0.3494	0.7927	0.087*	
H11B	0.1997	−0.3517	0.8556	0.087*	
C12	0.08232 (12)	−0.1303 (3)	0.90375 (11)	0.0604 (4)	
O1	0.07037 (12)	−0.2832 (3)	0.96952 (10)	0.0917 (5)	
H1	0.023 (2)	−0.225 (5)	1.011 (2)	0.138*	
O2	0.04415 (11)	0.0874 (2)	0.90046 (8)	0.0860 (5)	

### Atomic displacement parameters (Å^2^)

**Table:**

	*U*^11^	*U*^22^	*U*^33^	*U*^12^	*U*^13^	*U*^23^
C1	0.0591 (8)	0.0544 (9)	0.0523 (9)	0.0066 (7)	0.0167 (7)	−0.0096 (7)
C2	0.0627 (9)	0.0566 (9)	0.0557 (9)	0.0027 (7)	0.0183 (7)	−0.0095 (7)
C3	0.0734 (11)	0.0958 (14)	0.0620 (12)	−0.0017 (9)	0.0076 (9)	−0.0150 (9)
C4	0.0950 (14)	0.1195 (17)	0.0576 (11)	0.0153 (14)	0.0112 (10)	0.0128 (11)
C5	0.0952 (14)	0.0817 (13)	0.0698 (13)	0.0084 (11)	0.0328 (11)	0.0162 (10)
C6	0.0638 (9)	0.0585 (9)	0.0742 (11)	0.0034 (7)	0.0289 (8)	−0.0099 (8)
C7	0.0866 (13)	0.0785 (13)	0.1152 (19)	−0.0171 (11)	0.0461 (13)	−0.0290 (12)
C8	0.0819 (14)	0.133 (2)	0.126 (2)	−0.0281 (14)	0.0250 (14)	−0.0627 (18)
C9	0.0887 (14)	0.146 (2)	0.0808 (15)	−0.0005 (15)	−0.0074 (12)	−0.0414 (15)
C10	0.0830 (12)	0.0901 (13)	0.0600 (11)	0.0066 (10)	0.0102 (9)	−0.0117 (10)
C11	0.0857 (11)	0.0573 (10)	0.0774 (12)	−0.0064 (8)	0.0317 (9)	−0.0109 (8)
C12	0.0658 (9)	0.0517 (9)	0.0649 (10)	−0.0026 (7)	0.0202 (7)	0.0001 (7)
O1	0.1183 (11)	0.0755 (9)	0.0845 (9)	0.0278 (7)	0.0501 (8)	0.0218 (7)
O2	0.1095 (10)	0.0677 (8)	0.0838 (9)	0.0248 (7)	0.0461 (7)	0.0141 (6)

### Geometric parameters (Å, °)

**Table:**

C1—C10	1.413 (2)	C7—H7	0.9300
C1—C2	1.416 (2)	C8—C9	1.386 (4)
C1—C6	1.422 (2)	C8—H8	0.9300
C2—C3	1.365 (3)	C9—C10	1.360 (3)
C2—C11	1.500 (2)	C9—H9	0.9300
C3—C4	1.394 (3)	C10—H10	0.9300
C3—H3	0.9300	C11—C12	1.500 (2)
C4—C5	1.336 (3)	C11—H11A	0.9700
C4—H4	0.9300	C11—H11B	0.9700
C5—C6	1.405 (3)	C12—O2	1.2209 (19)
C5—H5	0.9300	C12—O1	1.2759 (19)
C6—C7	1.406 (3)	O1—H1	0.93 (3)
C7—C8	1.341 (4)		
			
C10—C1—C2	123.38 (16)	C6—C7—H7	119.4
C10—C1—C6	117.74 (16)	C7—C8—C9	120.3 (2)
C2—C1—C6	118.88 (15)	C7—C8—H8	119.8
C3—C2—C1	118.83 (15)	C9—C8—H8	119.8
C3—C2—C11	120.60 (16)	C10—C9—C8	121.0 (2)
C1—C2—C11	120.57 (15)	C10—C9—H9	119.5
C2—C3—C4	122.00 (19)	C8—C9—H9	119.5
C2—C3—H3	119.0	C9—C10—C1	120.6 (2)
C4—C3—H3	119.0	C9—C10—H10	119.7
C5—C4—C3	120.17 (19)	C1—C10—H10	119.7
C5—C4—H4	119.9	C12—C11—C2	114.62 (13)
C3—C4—H4	119.9	C12—C11—H11A	108.6
C4—C5—C6	121.07 (17)	C2—C11—H11A	108.6
C4—C5—H5	119.5	C12—C11—H11B	108.6
C6—C5—H5	119.5	C2—C11—H11B	108.6
C5—C6—C7	121.88 (18)	H11A—C11—H11B	107.6
C5—C6—C1	119.03 (16)	O2—C12—O1	122.64 (14)
C7—C6—C1	119.08 (19)	O2—C12—C11	122.59 (15)
C8—C7—C6	121.2 (2)	O1—C12—C11	114.75 (14)
C8—C7—H7	119.4	C12—O1—H1	114.4 (17)
			
C10—C1—C2—C3	−179.14 (15)	C2—C1—C6—C7	178.88 (14)
C6—C1—C2—C3	1.4 (2)	C5—C6—C7—C8	−179.27 (19)
C10—C1—C2—C11	0.8 (2)	C1—C6—C7—C8	0.5 (3)
C6—C1—C2—C11	−178.64 (12)	C6—C7—C8—C9	0.0 (3)
C1—C2—C3—C4	−0.4 (3)	C7—C8—C9—C10	−0.3 (4)
C11—C2—C3—C4	179.63 (16)	C8—C9—C10—C1	0.1 (3)
C2—C3—C4—C5	−0.6 (3)	C2—C1—C10—C9	−179.13 (17)
C3—C4—C5—C6	0.7 (3)	C6—C1—C10—C9	0.3 (2)
C4—C5—C6—C7	−179.91 (18)	C3—C2—C11—C12	−110.23 (19)
C4—C5—C6—C1	0.4 (3)	C1—C2—C11—C12	69.8 (2)
C10—C1—C6—C5	179.13 (15)	C2—C11—C12—O2	24.3 (3)
C2—C1—C6—C5	−1.4 (2)	C2—C11—C12—O1	−157.00 (17)
C10—C1—C6—C7	−0.6 (2)		

### Hydrogen-bond geometry (Å, °)

**Table:**

*D*—H···*A*	*D*—H	H···*A*	*D*···*A*	*D*—H···*A*
C3—H3···O2^i^	0.93	2.61	3.541 (2)	177
O1—H1···O2^ii^	0.93 (3)	1.76 (3)	2.6723 (17)	168 (3)
C11—H11B···Cg1^iii^	0.97	2.87	3.746 (2)	151

Symmetry codes: (i) −*x*, *y*−1/2, −*z*+3/2; (ii) −*x*, −*y*, −*z*+2; (iii) *x*, *y*−1, *z*.
